# Rural Health and Family Medicine: An Agenda for sub-Saharan Africa

**DOI:** 10.4102/phcfm.v3i1.271

**Published:** 2011-10-03

**Authors:** Emmanuel Monjok, Ita B. Okokon, Andrea Smesny, Ekere J. Essien

**Affiliations:** 1 Institute of Community Health, Texas Medical Center, University of Houston, United States; 2 Department of Family Medicine, College of Medical Sciences, University of Calabar, Nigeria

## To the editor:

The successful second Africa Regional WONCA (World Organization of Family Doctors) Conference (2009) that was held in Rustenburg, South Africa, was based on the theme of the role of the family physician in Africa. We are just as concerned as Dr Bernhard M. Gaede^1^ regarding the role of family physicians in rural health. We believe that extended procedural skills found amongst experienced district hospital generalist doctors should form part of the core skills for rural family physicians in Africa. Earlier in 2008, Disasters and Family Medicine, was the theme used in the Rural WONCA Conference in Calabar, Nigeria. Therefore, there is the concern that family physicians in Africa need the necessary knowledge to put them in advantaged positions to be able to effectively manage the medical problems that are common in their territories. 

It is common knowledge that most postgraduate medical training programmes in sub-Saharan Africa (SSA) tend to copy and follow the trend in Western Europe and North America. This should be prevented from occurring within family medicine. A recent article^2^ highlighted this fact and indicated that the design of family medicine as developed in high-income countries may not be applicable in SSA. The pattern of diseases, the health-manpower distribution, the shortages of other specialists (surgeons, obstetricians and gynaecologist, anaesthesiologists etc.) in the districts and the population in rural SSA, are quite different from those in high-income countries. These facts, therefore, need to be taken into consideration when designing family medicine training programmes in SSA.

Family medicine training in SSA should have an urban-based pathway as well as a rural-based pathway. The urban-based pathway would probably conform to what is obtained in advanced countries of Europe and North America. The rural-based family medicine training should be different and should include extended procedural skills especially for all procedures required in life-threatening medical and surgical conditions. The reasoning is based on the fact that it will take a very long time for all facets of development to reach the many underdeveloped districts where the majority of people reside and, by implication, where severe shortages of other medical specialists exist. The rural family medicine program for SSA should contribute to the development of the district health-care system, particularly in medical and health manpower. In this context, the training programme for rural family practice should include the skills of the district hospital generalist doctor (DHGD). A well planned and long-term training program should replace DHGDs with speciality-trained rural family physicians, until such a time as when the district hospitals in SSA have a sufficient number of hospital specialists in their employment. Eventually, the various experiences and interests of the rural family practitioners will lead to rural family physicians with experience in surgery, obstetrics, anaesthesiology, paediatric or child health, public health, primary health care, ophthalmology, mental health and so forth. This could lead to the introduction of shorter postgraduate training programmes in obstetrics^3^ and district hospital surgery^4^ in addition to those in public health, anaesthesiology, ophthalmology, psychiatry and ENT (ear, nose and throat) that already exist in some countries. This will lead to an effective coverage of both community-oriented primary care and the district hospital, which lacks the required hospital specialists in 90% of the districts in SSA. The postgraduate diploma programme in Family Medicine was successfully launched in October 2010 in Lagos, Nigeria, under the auspices of the Faculty of Family Medicine of the National Postgraduate Medical College of Nigeria, in collaboration with the Association of General and Private Medical Practitioners of Nigeria. It is against this background that we trust this postgraduate diploma programme in Family Medicine to address the peculiar rural problems of SSA and serve as a model for other developing and non-developed countries.

The concept of family physicians as specialists in primary care is one of providing community-oriented care with comprehensive and continuous care, targeting mainly chronic non-communicable diseases in an ever increasing elderly population as it occurs in high-income countries. This concept is completely different from the predominantly preventable diseases and trauma, with district hospitals severely lacking in other hospital specialists, as it occurs in SSA countries. This differences also calls for a different training strategy and approach for SSA. The Nigerian family medicine training program which started in 1980 with the combination of community-oriented primary care and extensive district hospital procedural skills, has been upgrading itself over the course of years. We believe that rural family medicine training in Africa should continue to include skills necessary for secondary health care at the district level until all developmental indices, including medical manpower, has engulfed the vast rural communities in these countries. Procedural task shifting to generalist doctors in SSA countries during medical and humanitarian missions by western super-specialists is a short term solution.^5,6^ The long term solution may involve planning for training:

- rural family physicians with additional training in obstetrics
- rural family physicians with additional training in district surgery
- rural family physicians with additional training in otolaryngology
- rural family physicians with additional training in ophthalmology
- rural family physicians with additional training in child health
- rural family physicians with additional training in mental health

Through distinct shorter training programmes suitable for low-income and resource-constrained countries of SSA. This idea should not be seen as reverting to the situation in which the family physician was perceived as a mini-specialist of every specialty with no domain of his or her own. The domain of family medicine in the SSA has after all already been defined,^7,8,9^ and this idea must be accepted as a very positive approach to solve the critical shortages of hospital specialists of these disciplines that will ensure that SSA countries make the necessary breakthrough before the end of this millennium. Training programmes are not static, and consequently this concept will change gradually over time, based on the epidemiological disease profile.

For this concept to occur, however, there has to be a high political priority and commitment to primary health care and district health care, as well as to medical and allied health-manpower development from the governments of SSA countries. This political commitment implies a well-designed career structure for rural health and family practice with financial benefits and incentives for family physicians and other rural health care workers. In addition, the training is designed for SSA and other similar countries in mind, and consequently it is anticipated that the success of these training programmes would lead to acceptance and recognition by medical societies in advanced nations with the ultimate effect of reducing physicians’ migration (brain drain) from Africa to Europe and North America.
